# Application of nafamostat mesylate in 5 cases of extracorporeal carbon dioxide removal combined with continuous renal replacement therapy

**DOI:** 10.3389/fphar.2025.1592562

**Published:** 2025-09-03

**Authors:** Ma Xiaobo, Chen Qiuxin, Yang Zhenhua, Pan Xiaoting, Chen Xiaonong

**Affiliations:** ^1^ Department of Nephrology, Shanghai Ruijin Hospital Affiliate to Shanghai Jiaotong University School of Medicine, Shanghai, China; ^2^ Department of Neurology and Nephrology, The Third Division Hospital of Xinjiang Production and Construction, Xinjiang, China

**Keywords:** extracorporeal carbon dioxide removal, anticoagulation, nafamostat mesylate, hypercapnia, CRRT

## Abstract

**Objective:**

To explore the application of nafamostat mesylate in the extracorporeal carbon dioxide removal combined with continuous renal replacement therapy.

**Method:**

We analyzed the cases of 5 patients who underwent extracorporeal carbon dioxide removal combined with continuous renal replacement therapy due to severe hypercapnia with acute kidney injury at the Blood Purification Center of Ruijin Hospital Affiliated to Shanghai Jiao Tong University School of Medicine from July 2023 to October 2024. The treatments were performed using the OMNI blood purification system (B. Braun Avitum AG, Melsungen, Germany), equipped with a PMP polymethylpentene membrane lung (1.81 m^2^, Eurosets Medolla, Italy), with unhumidified pure oxygen connected to the ECCO_2_R membrane lung via an oxygen supply device at a rate of 6–7 L/min, and in series with the blood filter (OMNIFilter 16, polyethersulfone membrane, 1.6 m^2^). CRRT was performed using the CVVH mode, with sodium lactate replacement fluid at a flow rate of 2–3 L/h. A 14Fr dual-lumen dialysis catheter (ARROW) was placed under ultrasound-guided puncture in the femoral vein. The extracorporeal blood flow was maintained between 300 and 400 mL/min. Continuous infusion of NM (50-30 mg/h) was used to maintain anticoagulation. Demographic and physiological data were collected (including vital signs, ventilation parameters, blood gas, electrolytes, DIC, etc.), with blood sampling points before and after the filter as well as peripheral blood.

**Results:**

Among the 5 patients, there were 2 males and 3 females, with an average age of 83.2 ± 9.6 years old. A total of 22 treatments were administered, with an average treatment time of 8.8 ± 1.6 h. All patients had primary diseases of severe pneumonia and chronic obstructive pulmonary disease; among them, 4 had combined renal injury, and 3 patients were on mechanical ventilation. During the total of 22 treatments, there was 1 instance of dialyzing tube occlusion which led to the end of treatment 2 h early, while the other 21 treatments were completed smoothly, with no occlusion occurring in the filter, ECCO_2_R membrane lung, or dialyzing tube. No adverse events such as bleeding occurred in the patients, and there were no statistically significant differences in APTT and PT after treatment compared to before. During the treatment, statistically significant improvements in PaCO_2_ and blood pH were observed. Blood pressure, heart rate, and blood oxygen saturation remained stable.

**Conclusion:**

Extracorporeal carbon dioxide removal combined with continuous renal replacement therapy can partially replace lung ventilation function and improve hypercapnia. NM can be used as an anticoagulant in this technology, with an ideal anticoagulation effect and no significant risk of bleeding.

## 1 Introduction

Extracorporeal carbon dioxide removal (ECCO_2_R) is a blood purification technique that removes carbon dioxide from the blood through a lower blood flow rate, thereby correcting hypercapnia and respiratory acidosis, mainly used for lung protective ventilation and treatment of hypercapnic respiratory failure in acute respiratory distress syndrome (ARDS) (Gattinoni et al., 1986). As a blood purification technique, anticoagulation is essential for ensuring the smooth progress of the treatment. Bleeding has been reported as one of the main adverse events of ECCO_2_R (Worku et al., 2023). Therefore, effective and safe anticoagulation is crucial. Nafamostat mesylate (NM) is currently being used more widely in China, but its application in ECCO_2_R has not been reported. This study aims to explore the use of NM in ECCO_2_R combined with continuous renal replacement therapy (CRRT).

## 2 Objects and methods

### 2.1 Research subject

Analysis of 5 patients who underwent extracorporeal carbon dioxide removal combined with continuous renal replacement therapy due to severe hypercapnia in the blood purification center of Shanghai Ruijin Hospital from July 2023 to October 2024.

### 2.2 Research methods

#### 2.2.1 Blood purification plan

All cases used ultrasound-guided percutaneous puncture technology to place a 14Fr double-lumen dialysis catheter (ARROW) in the femoral vein. Treatment was administered using the OMNI Blood Purification System (B.Braun Avitum AG, Melsungen, Germany), which was equipped with a PMP polymethylpentene membrane lung (1.81 m^2^, Eurosets Medolla, Italy). Unhumidified pure oxygen was connected to the ECCO_2_R membrane lung via an oxygen supply device at a rate of 6–7 L/min, and was placed in series before the blood filter (OMNIfilter 16, polyethersulfone membrane, 1.6 m^2^). CRRT was performed using the CVVH mode, with sodium lactate as the replacement fluid and a replacement fluid volume of 2–3 L/h. The extracorporeal blood flow was maintained at 300–400 mL/min, with a treatment duration of 8–12 h.

#### 2.2.2 Anticoagulation plan

Maintain extracorporeal anticoagulation was performed through continuous infusion of NM(50-30 mg/h), without loading dose. The initial dose was 50 mg/h at first. The dose was reduced to 40 mg/h at the 3rd hour and reduced to 30 mg/h at the 5th hour.

#### 2.2.3 Blood sample collection

Including blood routine (peripheral collection before and after treatment), blood gas, electrolytes (peripheral collection before and after treatment, and collection before the pump of NM during treatment), DIC (peripheral collection before and after treatment, hourly collection after the filter during the first treatment of cases 1, 2, and 4). The time point for blood collection after treatment was 10 min post-therapy.

### 2.3 Observation index

#### 2.3.1 Baseline datas

Including age, sex, primary disease, ventilation status, vital signs, hemoglobin, platelets, prothrombin time (PT), activated partial thromboplastin time (APTT), blood pH value, oxygen partial pressure, carbon dioxide partial pressure, bicarbonate concentration.

#### 2.3.2 Dialyzer clotting situation

Clotting assessment: Level 0: No clotting or a few fibrin clots; Level I: Less than 10% fibrin clots or clustered fibrin clots; Level II: Less than 50% fibrin clots or severe clotting; Level III: More than 50% fibrin clots, significantly elevated venous pressure, or requires replacement of the dialyzer (Zhuang et al., 2022).

#### 2.3.3 Vital signs, laboratory tests

The patients’ vital signs, APTT, PT, blood pH value, oxygen partial pressure, carbon dioxide partial pressure, and bicarbonate concentration before, during, and after treatment.

#### 2.3.4 Adverse events

Including adverse events such as bleeding, allergies, arrhythmias, and hyperkalemia.

### 2.4 Statistical methods

Data processing and analysis were performed using SPSS 26.0 software. Continuous variables were described using mean ± standard deviation (x ± s), and categorical variables were described using cases (percentage) [n (%)]. Wilcoxon signed-rank test were used for comparisons between two groups. Cases 1, 2, and 4 underwent normal distribution testing for APTT and PT during the first treatment, correlation tests were conducted on the two sets of data, and the trend of changes was analyzed using the Mann-Kendall trend test. A difference was considered statistically significant at P < 0.05.

## 3 Results

### 3.1 Baseline and treatment status

Among the 5 patients, there were 2 males and 3 females, with an average age of 83.2 ± 9.6 years old. All patients had primary diseases of severe pneumonia and chronic obstructive pulmonary disease (COPD); all patients had acute kidney injury, the creatinine levels of 4 patients increased, and 3 patients required mechanical ventilation. A total of 22 treatments were administered, with an average treatment time of 8.8 ± 1.6 h. During the total of 22 treatments, there was 1 instance of dialyzing tube occlusion which led to the end of treatment 2 h early, while the other 21 treatments were completed successfully. (See Table 1 for details).

**TABLE 1:** Baseline and treatment conditions.

Case number	Sex	Age	Primary disease	Ventilation status	No. of treatment	Early finish	Time of treatment (h/scene)	Blood flow (mL/min)	Gas flow (L/min)	Prognosis
Case 1	F	92	Pneumonia, COPD	High flow	1	0	8	300	6	improved
Case 2	M	76	Pneumonia, COPD	mechanical ventilation	1	1	10	300–370	7	dead
Case 3	M	89	Pneumonia, COPD	mechanical ventilation	7	0	9.1 ± 2.0	370–400	7	dead
Case 4	F	70	Pneumonia, COPD	mechanical ventilation	3	0	8	370	6	dead
Case 5	F	89	Pneumonia, COPD	BiPAP	10	0	8.8 ± 1.7	300–350	6	improved

### 3.2 Blood gas monitoring status

During the treatment period, the blood gas results (pH value, partial pressure of oxygen, partial pressure of carbon dioxide, oxygen saturation, bicarbonate concentration) of all cases showed statistically significant improvements. (See Table 2 for details).

**TABLE 2:** Blood gas monitoring results before and after treatment.

Case number	Scene	PH		PO_2_(kPa)		PCO_2_(kPa)		SO_2_(%)		HCO_3_−(mmol/L)	
		Before	After	Before	After	Before	After	Before	After	Before	After
Case 1	1	7.32	7.41	8.71	15.12	8.59	7.35	91.7	98.2	28.7	31.4
Case 2	1	7.32	7.39	12.31	19.33	7.79	7.64	93.1	98.5	24.3	26.8
Case 3	1	7.19	7.33	-	-	9.08	8.87	98.2	98.3	21.6	21.2
	2	7.28	7.27	10.50	10.30	6.97	7.07	98.3	94.6	21.7	24.0
	3	7.33	7.36	11.20	11.60	6.67	6.40	98.3	98.5	22.1	22.7
	4	7.36	7.34	12.10	11.90	6.40	6.67	98.4	98.3	24.0	24.0
	5	7.25	7.27	8.12	9.57	6.80	6.53	91.0	94.3	22.0	23.0
	6	7.31	7.35	10.80	12.20	5.87	5.07	93.2	96.1	23.0	25.0
	7	7.35	7.41	9.60	13.87	5.87	4.53	95.5	98.7	24.0	25.0
Case 4	1	7.05	7.17	10.10	12.49	10.56	7.50	87.8	94.6	17.2	20.5
	2	7.20	7.34	10.62	13.47	7.93	5.32	93.4	96.2	20.1	22.3
	3	7.31	7.40	13.72	15.32	7.12	5.01	95.2	97.3	21.4	25.3
Case 5	1	7.21	7.28	14.12	15.07	8.01	7.64	94.3	96.2	21.4	22.3
	2	7.24	7.32	13.89	14.01	7.94	7.32	95.1	95.0	20.8	23.1
	3	7.29	7.37	15.08	15.11	7.34	6.92	96.3	97.1	21.3	24.4
	4	7.25	7.34	14.4	14.8	7.95	7.21	96.3	96.2	20.7	23.1
	5	7.28	7.36	14.3	14.2	7.61	7.32	97.1	97.4	20.4	24.2
	6	7.31	7.40	15.4	15.32	7.33	6.98	96.1	97.2	22.5	25.1
	7	7.32	7.39	15.13	15.16	6.81	6.59	98.1	97.6	23.3	25.6
	8	7.35	7.41	15.57	15.34	6.61	6.28	97.2	98.4	23.3	26.4
	9	7.37	7.42	14.98	14.6	6.32	5.98	98.3	98.5	23.5	25.8
	10	7.37	7.41	15.01	14.9	6.11	5.46	97.3	98.1	24.0	26.1
P Value		<0.001		0.016		<0.001		0.003		<0.001	

### 3.3 Coagulation indicators and blood routine monitoring

There were no statistically significant differences in APTT, PT, FIB, DD, and hemoglobin before and after all 22 treatments, the platelets before and after treatments were 148.0 ± 58.0 vs 145.1 ± 55.8 (P = 0.047) (peripheral collection, see Table 3).

**TABLE 3:** APTT, PT, FIB, D-Dimer, PLT, HB before and after treatment (peripheral collection).

Case number	APTT(s)		PT(s)		FIB(g/L)		D-Dimer (ug/mL)		Plt (*10^9^/L)		Hb(g/L)	
	Before	After	Before	After	Before	After	Before	After	Before	After	Before	After
Case 1	27.80	26.80	11.50	11.10	4.30	3.60	1.13	1.22	120	114	98	101
Case 2	35.00	33.60	12.70	11.80	5.20	5.10	0.92	1.04	212	199	84	80
Case 3	32.60	34.00	11.70	12.20	5.70	5.10	5.55	6.12	101	105	92	90
	33.70	33.40	12.00	11.70	4.90	4.70	6.01	6.20	93	95	89	93
	31.20	34.20	11.50	12.70	4.50	4.40	5.97	6.10	105	100	91	88
	33.40	32.90	12.50	11.80	5.10	5.30	5.60	5.30	94	90	87	87
	31.50	32.50	11.80	12.40	4.20	4.00	5.80	5.91	86	89	84	82
	32.60	33.40	12.30	12.40	4.50	5.10	5.32	5.10	88	82	87	90
	32.70	32.30	12.30	12.00	5.10	5.20	6.13	6.20	91	89	91	93
Case 4	28.60	28.50	11.10	11.30	3.40	3.40	3.18	2.98	78	83	75	71
	29.40	29.20	11.80	11.40	3.20	3.10	2.87	2.60	81	79	72	73
	32.40	33.50	12.00	12.50	3.50	3.20	2.90	3.10	85	80	74	72
Case 5	39.3	37.3	12.4	12	4.20	3.80	1.20	0.97	213	204	109	106
	38.6	38.9	12.2	11.9	3.90	3.97	1.10	0.90	224	217	105	107
	35.8	36.2	11.8	12.3	3.40	3.20	0.80	0.91	209	198	110	106
	35.6	34.8	11.9	12	3.50	3.20	1.09	1.18	186	193	106	103
	33.4	34.2	10.8	11.5	3.40	3.40	1.20	1.08	194	203	103	105
	36.20	36.60	11.8	12.1	3.60	3.50	1.30	1.13	214	204	107	103
	34.2	34.7	11.2	11.9	3.10	2.90	1.15	1.08	200	191	109	107
	31.2	32.4	10.7	11.3	3.20	3.40	0.97	0.87	196	199	105	107
	32.4	33.3	11.7	12.3	3.60	3.80	1.12	1.30	191	190	110	106
	34.2	33.4	12.2	11.1	3.70	3.60	1.10	1.05	194	189	107	104
P Value	0.389		0.435		0.071		0.638		0.047		0.085	

We monitored the hourly peripheral APTT and PT values for Case 1, which remained within the normal range and showed significant differences from the post-filter results. Considering the blood volume drawn, we did not perform this test during treatment in the remaining patients. (see Table 4).

**TABLE 4:** Hourly APTT and PT results of case 1.

Time of collection	APTT(s)		PT(s)	
	Peripheral collection	Collection after the filter	Peripheral collection	Collection after the filter
Before	27.80	–	11.50	–
1 h	28.30	103.30	12.40	13.20
2 h	28.00	100.80	12.30	13.80
3 h	28.40	103.30	12.60	14.90
4 h	29.50	109.90	12.80	14.30
5 h	32.30	113.70	13.10	16.20
6 h	30.40	108.40	12.50	15.10
7 h	31.20	109.00	13.20	17.40
After	26.80	–	11.10	–

**FIGURE 1:**
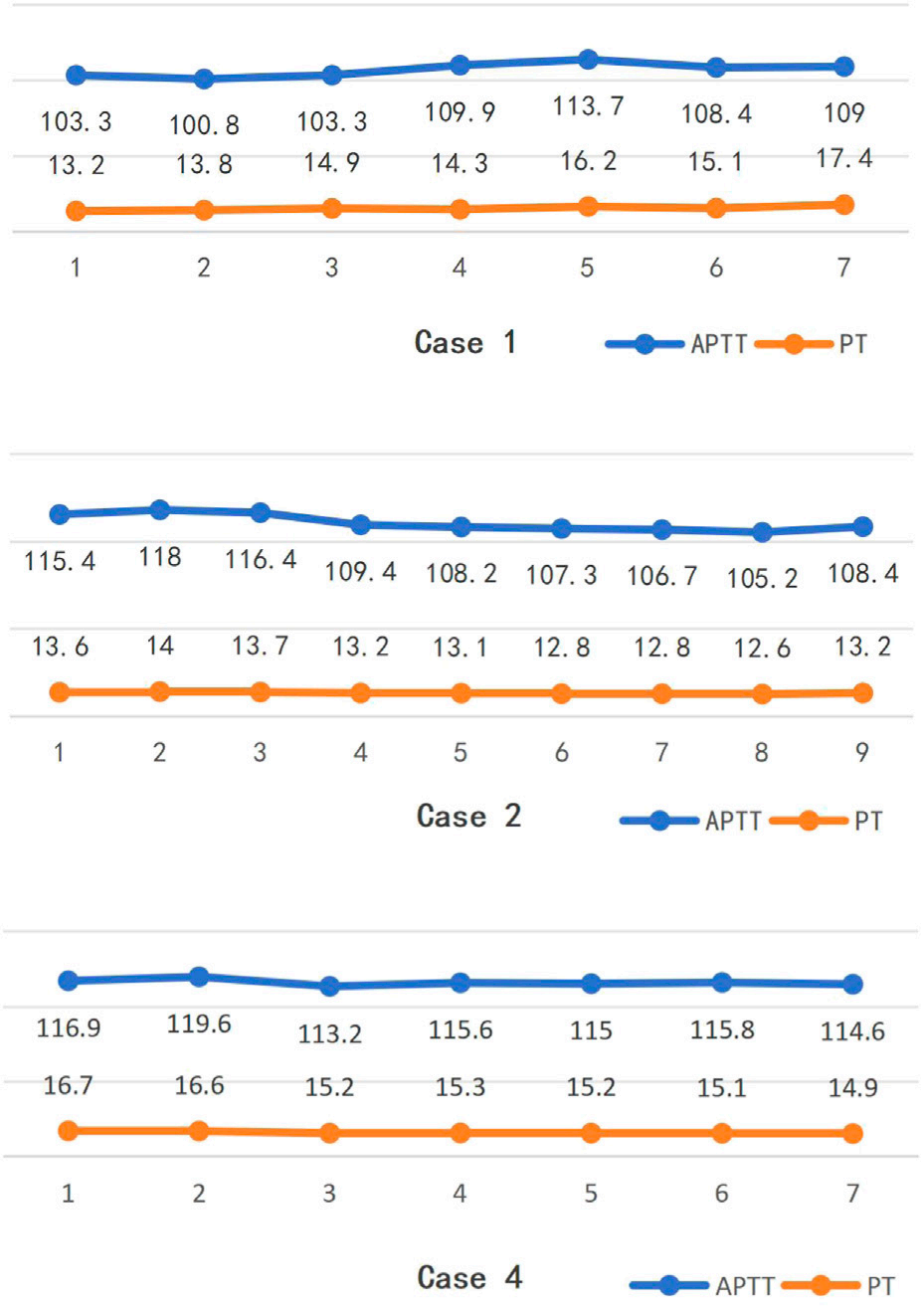
Changes trends in APTT and PT during the 1st treatment of some cases (collection after the filter).

In cases 1, 2, and 4, the changes in APTT and PT during the first treatment are shown in Figure 1 (collection after the filter). In case 1, the average APTT was (106.5 ± 4.6) s and the average PT was (14.7 ± 1.6) s; the Mann-Kendall test showed that the Spearman rank correlation coefficient for APTT was r_s_ = 0.50, P = 0.12, and for PT, it was r_s_ = 0.82, P = 0.02. In case 2, the average APTT was (111.3 ± 5.1) s and the average PT was (13.2 ± 0.5) s; the Mann-Kendall test showed that the Spearman rank correlation coefficient for APTT was r_s_ = −0.92, P = 0.001, and for PT, it was r_s_ = −0.83, P = 0.01. In case 4, the average APTT was (115.1 ± 2.2) s and the average PT was (15.6 ± 0.7) s; the Mann-Kendall test showed that the Spearman rank correlation coefficient for APTT was r_s_ = −0.14, P = 0.65, and for PT, it was r_s_ = −0.93, P = 0.01. APTT and PT were normally distributed, with a Pearson correlation coefficient of 0.483, P = 0.019.

### 3.4 Dialyzer clotting situation

Judgment of dialyzer clotting: Grade 0 in 2 cases (9.1%), Grade I in 16 cases (72.7%), Grade II in 3 cases (13.6%), Grade III in 1 case (4.5%), with an effective anticoagulation rate of 81.8% (Grades 0–1).

### 3.5 Safety

During the 22 treatments, one patient experienced once a deepening of the color of the chest drainage fluid, which improved the next day after treatment, with no other active bleeding occurrences. All patients maintained stable vital signs throughout the treatment process, with no instances of hypotension or arrhythmia. No adverse events such as allergies or hyperkalemia occurred.

## 4 Discussion

Since the COVID-19 pandemic, extracorporeal membrane oxygenation (ECMO) technology has gradually received widespread attention and application. However, ECMO has a high technical threshold, and its invasive and complex characteristics lead to a higher risk of complications (Mi et al., 2018), making it difficult for ordinary blood purification centers to promote. The principle of ECCO_2_R is similar to that of ECMO; although its oxygenation capacity is lower, it effectively removes carbon dioxide from the body (Ding et al., 2021). Therefore, it can achieve a “protective” ventilation strategy for patients in acute and severe decompensation stages of acute respiratory distress syndrome (ARDS) and chronic obstructive pulmonary disease (COPD), alleviating the effects of hypercapnia and acidosis (Zhang et al., 2023). At the same time, due to the high capacity of blood to carry carbon dioxide, the extracorporeal circulation blood flow required for removal is relatively low (de Villiers Hugo et al., 2017), making the technical difficulty less than that of ECMO, thus leading to its increasing clinical applications.

As a type of extracorporeal circulation life support therapy, adequate anticoagulation is essential for ECCO_2_R treatment. Reference reports that hemorrhage, thrombocytopenia, and hemolysis are the main complications of ECCO_2_R (Worku et al., 2023; Kalbhenn et al., 2017). How to achieve effective anticoagulation to ensure treatment efficacy without increasing the risk of complications is a challenge that we must answer. Although the blood flow rate of ECCO_2_R is lower than that of ECMO, it is still higher than that of standard CRRT treatment. Using citrate anticoagulation may lead to excessive dosing and increase the risk of complications, therefore, we currently mainly use sodium heparin for systemic anticoagulation (Combes et al., 2020). Considering that patients undergoing ECCO_2_R treatment are often critically ill and have a higher risk of bleeding compared to regular patients, heparin anticoagulation may also lead to heparin-induced thrombocytopenia and other issues (Giraud et al., 2021), thus systemic heparin anticoagulation might not be the most ideal choice.

To address this question, we used NM for anticoagulation in 5 patients undergoing ECCO_2_R treatment in our center. Our center was among the early adopters of anticoagulation therapy using NM. Throughout our clinical experience, NM has demonstrated satisfactory safety and efficacy profiles. Therefore, for these five patients, we opted for NM anticoagulation to mitigate bleeding risks. NM is a broad-spectrum, potent serine protease inhibitor (Willers et al., 2021; Hwang et al., 2013); it has a short half-life of only 8 min *in vivo*, making it less likely to accumulate in the body (Song et al., 2022), and it is suitable for use in patients at high risk of bleeding (Yu et al., 2015). In this study, none of the 22 patients showed statistically significant changes in DIC and hemoglobin before and after treatment, with only one case of deepening color in the pleural drainage fluid. The adverse event rate was 4.5%, with no active bleeding occurring. Although the P-value for platelets before and after treatment was <0.05, the difference between the two means was small, with both values remaining within the normal range. So it shows the safety of NM in ECCO_2_R treatment. Future studies with larger sample sizes are required for further validation. The anticoagulation efficacy of the dialyzer (Grade 0–1) was 81.8%, with only 1 case of premature circuit clotting among the 22 treatments. The treatment completion rate was 95.5%. Simultaneously, the blood pH, oxygen partial pressure, carbon dioxide partial pressure, oxygen saturation, and bicarbonate concentration all improved with statistically significant differences. It shows the anticoagulant efficacy of NM in ECCO_2_R treatment and the effectiveness of ECCO_2_R treatment in patients with severe pneumonia and COPD.

We had taken hourly collection after the filter during the first treatment of cases 1, 2, and 4 to monitor the APTT and PT. The values of APTT and PT are correlated, indicating that NM has a certain effect on both. However, the increase in APTT is significantly higher than that of PT, thus anticoagulation monitoring should focus on APTT (Maruyama et al., 2011; Park et al., 2015). The trends of APTT in the three patients during treatment were first increasing then decreasing, decreasing, and decreasing respectively. But only the trend in the second case was statistically significant, and none showed an increasing trend. This indicates that NM metabolizes quickly and does not accumulate over time. Considering that the anticoagulation protocols were the same for all three, it also suggests that the correlation between NM dosage and APTT is not clear (Lim et al., 2016). Additionally, we monitored the first patient’s peripheral APTT and PT hourly, with all values remaining within normal ranges. This finding further indicates the safety profile of NM anticoagulation therapy. When using systemic heparin for anticoagulation, it is recommended to extend APTT to 1.5–2 times of baseline or maintain it between 45–60 s (Staudinger, 2020). In our study, the APTT after filters was 3–4 times of baseline, which is significantly different from the reference. But the reference did not specify the location of the blood sampling, which might be one reason for the difference.

Our study has several limitations. Due to the high cost of ECCO_2_R therapy and its restricted patient eligibility criteria, our sample size remained limited. Consequently, the extent to which our findings are representative in broader clinical contexts requires verification through larger-scale studies and multicenter investigations. Moreover, the constrained sample size, coupled with our center’s non-implementation of citrate anticoagulation, precluded comparative analyses with alternative anticoagulation strategies.

In summary, Nafamostat Mesilate holds considerable promise as an emerging anticoagulant option for ECCO_2_R, demonstrating favorable safety and efficacy profiles. However, further research is needed to answer the specific dosage, target values of monitoring indicators, monitoring protocols, and blood sampling locations.

## Data Availability

The original contributions presented in the study are included in the article/supplementary material, further inquiries can be directed to the corresponding author.

## References

[B1] Combes A. Auzinger G. Capellier G. du Cheyron D. Clement I. Consales G. (2020). ECCO2R therapy in the ICU: consensus of a European round table meeting. Crit. Care 24 (1), 490. 10.1186/s13054-020-03210-z 32768001 PMC7412288

[B2] de Villiers Hugo J. Sharma A. S. Ahmed U. Weerwind P. W. (2017). Quantification of carbon dioxide removal at low sweep gas and blood flows. J. Extra Corpor. Technol. 49 (4), 257–261. 10.1051/ject/201749257 29302116 PMC5737416

[B3] Ding X. Chen H. Zhao H. Zhang H. He H. Cheng W. (2021). ECCO2R in 12 COVID-19 ARDS patients with extremely low compliance and refractory hypercapnia. Front. Med. (Lausanne) 8, 654658–658. 10.3389/fmed.2021.654658 34307397 PMC8295461

[B4] Gattinoni L. Pesenti A. Mascheroni D. Marcolin R. Fumagalli R. Rossi F. (1986). Low-frequency positive-pressure ventilation with extracorporeal CO2 removal in severe acute respiratory failure. JAMA, 256(7), 881–886. 10.1001/jama.1986.03380070087025 3090285

[B5] Giraud R. Banfi C. Assouline B. De Charrière A. Cecconi M. Bendjelid K. (2021). The use of extracorporeal CO2 removal in acute respiratory failure. Ann. Intensive Care 11 (1), 43. 10.1186/s13613-021-00824-6 33709318 PMC7951130

[B6] Hwang S. D. Hyun Y. K. Moon S. J. Lee S. C. Yoon S. Y. (2013). Nafamostat mesilate for anticoagulation in continuous renal replacement therapy. Int. J. Artif. Organs 36(3): 208–216. 10.5301/IJAO.5000191 23404639

[B7] Kalbhenn J. Neuffer N. Zieger B. Schmutz A. (2017). Is extracorporeal CO2 removal really “Safe” and “Less” invasive? Observation of blood injury and coagulation impairment during ECCO2R. ASAIO J. 63(5): 666–671. 10.1097/MAT.0000000000000544 28187047

[B8] Lim J. Y. Kim J. B. Choo S. J. Chung C. H. Lee J. W. Jung S. H. (2016). Anticoagulation during extracorporeal membrane oxygenation; nafamostat mesilate *versus* heparin. Ann. Thorac. Surg. 102 (2), 534–539. 10.1016/j.athoracsur.2016.01.044 27083248

[B9] Maruyama Y. Yoshida H. Uchino S. Yokoyama K. Yamamoto H. Takinami M. (2011). Nafamostat mesilate as an anticoagulant during continuous veno-venous hemodialysis: a three-year retrospective cohort study. Int. J. Artif. Organs 34 (7), 571–576. 10.5301/IJAO.2011.8535 21786254

[B10] Mi M. Y. Matthay M. A. Morris A. H. (2018). Extracorporeal membrane oxygenation for severe acute respiratory distress syndrome. N. Engl. J. Med. 379 (9), 884–887. 10.1056/NEJMclde1804601 30157406

[B11] Park J. H. Her C. Min H. K. Kim D. K. Park S. H. Jang H. J. (2015). Nafamostat mesilate as a regional anticoagulant in patients with bleeding complications during extracorporeal membrane oxygenation. Int. J. Artif. Organs 38 (11), 595–599. 10.5301/ijao.5000451 26728787

[B12] Song J. Zhang W. Zhang L. Yang J. Zhang J. Zhou J.(2022). Standardized assessment of coagulation dysfunction in critically ill patients: a consensus among Chinese experts. J. People Liberation Army Med. Corps 47(2): 107–117. 10.11855/j.issn.0577-7402.2022.02.0107

[B13] Staudinger T. (2020). Update on extracorporeal carbon dioxide removal: a comprehensive review on principles, indications, efficiency, and complications. Perfusion 35 (6), 492–508. 10.1177/0267659120906048 32156179

[B14] Willers A. Arens J. Mariani S. Pels H. Maessen J. G. Hackeng T. M. (2021). New trends, advantages and disadvantages in anticoagulation and coating methods used in extracorporeal life support devices. Membr. (Basel) 11(8):617, 10.3390/membranes11080617 34436380 PMC8399034

[B15] Worku E. Brodie D. Ling R. R. Ramanathan K. Combes A. Shekar K. (2023). Venovenous extracorporeal CO2 removal to support ultraprotective ventilation in moderate-severe acute respiratory distress syndrome: a systematic review and meta-analysis of the literature. Perfusion 38 (5), 1062–1079. 10.1177/02676591221096225 35656595

[B16] Yu G. Li S. Wan R. Wang X. Hu G. (2015). Nafamostat mesilate for prevention of post-ERCP pancreatitis: a meta-analysis of prospective, randomized, controlled trials. Pancreas 44(4): 561–569. 10.1097/MPA.0000000000000310 25822153

[B17] Zhang L. Mingpeng L (2023). Advances in the clinical application of extracorporeal carbon dioxide removal technology. J. Southwest Med. Univ. 46(5): 369–373. 10.3969/j.issn.2096-3351.2023.05.001

[B18] Zhuang B. Ye H. Cao H. (2022). Multicenter randomized controlled study of injections of methylsulfonylmethane for anticoagulation treatment in hemodialysis. Chin. J. Blood Purif. 21(10): 739–743. 10.3969/j.issn.1671-4091.2022.10.008

